# Demographic and seasonal characteristics of respiratory pathogens in neonates and infants aged 0 to 12 months in the Central‐East region of Tunisia

**DOI:** 10.1002/jmv.25347

**Published:** 2018-11-21

**Authors:** Ines Brini Khalifa, Naila Hannachi, Aida Guerrero, Dorothea Orth‐Höller, Sana Bhiri, Jihene Bougila, Lamia Boughamoura, Sonia Nouri Merchaoui, Hassen Sboui, Nabiha Mahdhaoui, Britta Schiela, Dorothee Holm‐von Laer, Jalel Boukadida, Heribert Stoiber

**Affiliations:** ^1^ Department of Microbiology, Immunology, and Parasitology, Laboratory of Microbiology, Sousse Medical University, University of Sousse Sousse Tunisia; ^2^ Department of Microbiology, Research Unit for Genomic Characterization of Infectious Agents UR12SP34, University‐Hospital of Farhat Hached of Sousse Sousse Tunisia; ^3^ Doctoral School of Biological Sciences, Biotechnology and Health, Higher Institute of Biotechnology of Monastir, Faculty of Pharmacy of Monastir, University of Monastir Monastir Tunisia; ^4^ Division of Virology, Innsbruck Medical University Innsbruck Austria; ^5^ Division of Hygiene and Medical Microbiology Innsbruck Medical University Innsbruck Austria; ^6^ Department of Epidemiology and Medical Statistics, Division of Medical Statistics, University‐Hospital of Farhat Hached of Sousse Sousse Tunisia; ^7^ Pediatric Ward, University‐Hospital of Farhat Hached of Sousse Sousse Tunisia; ^8^ Neonatology Ward, University‐Hospital of Farhat Hached of Sousse Sousse Tunisia

**Keywords:** demography, infants, molecular assays, neonates, respiratory agents, seasonality

## Abstract

**Background:**

This study aimed to characterize the epidemiology of pathogenic respiratory agents in patients aged 0 to 12 months and hospitalized for acute respiratory infections in Tunisia between 2013 and 2014.

**Methods:**

A total of 20 pathogens, including viruses, *Mycoplasma pneumoniae*, and *Streptococcus pneumoniae*, were detected using molecular sensitive assays, and their associations with the patient’s demographic data and season were analyzed.

**Results:**

Viral infectious agents were found in 449 (87.2%) of 515 specimens. Dual and multiple infectious agents were detected in 31.4% and 18.6% of the samples, respectively. Viral infection was predominant in the pediatric environment (90.8%, *P* < 0.001), male patients (88.0%), and spring (93.8%). *Rhinovirus* was the most detected virus (51.8%) followed by *respiratory syncytial virus A/B* (34.4%), *coronavirus group* (18.5%), *adenovirus* (17.9%), and *parainfluenza viruses* 1‐4 (10.9%). Respiratory Syncytial virus A/B was significantly associated with gender (38.0% male cases vs 28.3% female cases, *P* = 0.02). Infections by *Adenovirus*, *Bocavirus*, and *Metapneumovirus A/B* increased with increasing age of patients (predominated cases aged 6‐12 months, *P* < 0.001). *S. pneumoniae* was detected in 30.9% of th tested samples. In 18.2% of the negative viral infections, only *S. pneumoniae* was identified.

**Conclusion:**

A predominance of the rhinovirus infection was observed in this study. Coronavirus subtypes were described for the first time in Tunisia. The observed different pathogenic profiles across age groups could be helpful to avoid the misclassification of patients presenting with ARIs at the triage level when no standardized protocol is available. This study will provide clues for physicians informing decisions regarding preventive strategies and medication in Tunisia.

## Abstract (highlights)

This study provides a useful starting point for a better understanding about the circulation of respiratory infectious agents in the area of Sousse, Tunisia. It will participate to reduce the overuse of unnecessary antibiotics in public hospitals which will avoid bacterial resistance and contribute to the implementation of preventive strategies and control systems in Tunisia.

## 1 INTRODUCTION

The World Health Organization estimates that acute respiratory infections (ARIs) are the second leading cause of childhood death, and about 70% of these infections occur in Africa and Southeast Asia.^1^ ARIs are responsible for 20% to 40% of children hospitalizations, and their course of infection is more severe in children aged less than 5 years.^2^

ARIs are caused by a variety of pathogenic infectious agents including viruses, bacteria, and fungi, of which viruses are the most predominant. The viruses associated with ARIs are classified as “old respiratory viruses” and “novel respiratory viruses.” The “old respiratory viruses” include *respiratory syncytial virus A/B* (RSV A/B); *influenza virus A* and *influenza virus B* (InfV‐A, InfV‐B); *parainfluenza virus 1‐3* (PIVs 1‐3); *adenovirus* (AdV); and *rhinovirus* (RV).^3^, ^4^ The “novel respiratory viruses” were identified more recently and comprise *metapneumovirus A/B* (MPV A/B), *severe acute respiratory syndrome coronavirus, coronavirus NL63*, *coronavirus HKU1* , *coronavirus OC43*, *coronavirus 229E* (CoV‐NL63, CoV‐HKU1, CoV‐OC43, and CoV‐229E), PIV‐4, and *bocavirus* (BoV). In recent years, more respiratory viruses, such as enterovirus genus (EV), parechovirus genus (PeV), InfV‐A *(H1N1)*, and *influenza virus C* , have appeared more frequently as the cause of ARIs.^5^ In addition to viral infections, bacteria like *Mycoplasma pneumoniae* (*M. pneumoniae*) and *Streptococcus pneumoniae* (*S. pneumoniae*) have been the most associated bacteria with ARIs.^6^ Fungal ARIs in children are rare and are found mainly in immunocompromised patients.^7^

The acquisition and spreading of ARIs vary among study populations in different countries. This may be due to children’s malnutrition, the low socioeconomic status of the country, cultural habits in the community, patient’s immune deficiency, local/regional geographical and climate change, and the variability of the health care systems in public and private hospitals. Consequently, the morbidity related to ARIs is mostly observed in the developing world.^8^ On the other hand, the expanding number of pathogens identified as a causative agent of ARIs might be due to the introduction of molecular techniques such as the multiplex real‐time polymerase chain reactions (qPCR, qRT‐PCR) in routine. These assays have increased the sensitivity and specificity of detection and allow the identification of a wide range of viruses and bacteria in one reaction. This will be helpful for the better understanding of the pathogenesis of ARIs.^9^ However, in most developing countries, the application of multiplex assays has not been fully implicated in the routine laboratory testing due to their high costs. As far as the Arab Maghreb is concerned, laboratories perform mostly enzyme immunoassays, rapid diagnostic tests, or latex agglutination techniques for some frequent pathogens, but no routine tests using qPCR assays are generally available. Owing to the absence of detailed diagnostic testing, infections are managed empirically by antibiotic regimens, resulting in an overuse of broad‐spectrum antibiotics, favoring the development of bacteria (bacterial resistance).^10^

In Tunisia, studies on the etiology of a large range of respiratory microbes across the seasons, among various ages and genders—and with the application of molecular and sensitive assays—are limited. A Tunisian study published by el Moussi et al in 2013 reports the distribution of the most common respiratory viruses using a multiplex assay. However, no association between viral infection and sociodemographic situation of patients or the seasonal distribution of detected pathogens was observed by this study. In addition, bacterial identification was excluded.^11^

In light of this, in the present study, a total of 18 viruses associated with ARIs were analyzed using a sensitive multiplex molecular assay. In addition, detection of the bacterial genomes *M. pneumoniae* and *S. pneumoniae* was performed. Moreover, the demographic characteristics of the detected pathogens, as well as their seasonality, were analyzed. This study aimed to gain a better understanding of the epidemiology of the most common respiratory microbes in the area of Sousse, Tunisia. Such findings will help to reduce the use of unnecessary antibiotics, thus, avoiding bacterial resistance and contributing to the implementation of prevention and control systems in Tunisia.

## 2 MATERIAL AND METHODS

### 2.1 Ethics approval

The present study was approved with a formal authorization by the Scientific and Ethical Committee of Farhat Hached University‐Hospital (FH‐UH) of Sousse, Tunisia. The approval number is IRB 00008931, provided by OHRP.

### 2.2 Subjects

A total of 515 subjects aged 0 to 12 months and hospitalized for upper and/or lower ARIs in the pediatric (PP) and the neonatology (NN) wards of FH‐UH of Sousse, Tunisia, were enrolled. Demographic data, including age, ward of admission, date of hospitalization, season, and gender, were obtained using a full‐filled information form. The study population was divided into three age groups. The first group G1 included neonates aged 0 to 28 days and the second and third groups contained infants. The groups of infants were described as follows: group G2 was characterized by patients aged 28 days to 6 months, and the third group G3 comprised subjects aged 6 to 12 months (Table 1).

**Table 1:** Demographic characteristics of neonates and infants hospitalized in the neonatology and pediatric wards of Farhat Hached University‐hospital of Sousse, Tunisia between September 2013 and December 2014

Patient's characteristics		Total population	Neonatology ward	Pediatric ward	
Age groups		No. (%)	G1 (0‐28 d)	G2 (28 d‐6 mo)	G3 (6‐12 mo)
Total subjects		515 (100.0)	211 (41.0)	206 (40.0)	98 (19.0)
Gender	Male	324 (62.9)	123 (58.3)	136 (66.0)	65 (66.3)
	Female	191 (37.1)	88 (41.7)	70 (34.0)	33 (33.7)
Season	Sep‐Dec 2013	160 (31.0)	93 (44.1)	41 (19.9)	26 (26.5)
	Jan‐Mar 2014	238 (46.2)	89 (42.2)	107 (51.9)	42 (42.9)
	Apr‐Jun 2014	65 (12.6)	20 (9.5)	30 (14.5)	15 (15.3)
	Jul‐Sep 2014	18 (3.5)	2 (1.0)	11 (5.3)	5 (5.1)
	Oct‐Dec 2014	34 (6.6)	7 (3.3)	17 (8.2)	10 (10.2)

Abbreviations: G1, age group 1; G2, age group 2; G3, age group 3; No., number, (%): percentage.

The percentages were calculated dividing the number of subjects defined by each category/group by the total number of cases belonging to each column: 515 enrolled subjects (column “Total population”), 211 neonates (G1 age group), and 304 infants including 206 cases in the G2 age group and 98 subjects belonging to the G3 age group.

### 2.3 Study design

This is a retrospective, descriptive, and analytic study carried out from September 31, 2013 to December 31, 2014 (October 2013‐December 2014) with a collaboration between the Ministry of Higher Education and Scientific Research of Tunisia (Unit Research for Genomic Characterization of Infectious Agents, UR12SP34, FH‐UH of Sousse and Laboratory of Microbiology, Sousse Medical University, Tunisia) and Innsbruck Medical University, Austria (Division of Virology and Division of Hygiene and Medical Microbiology). Sampling and rapid diagnostic assays using the direct/indirect immunofluorescence and/or immunochromatography assays for selected viruses (data and results are not shown) were carried out in Tunisia. Nasopharyngeal aspirates were obtained at the time of admission and were transferred on the same day to the laboratory of Microbiology. The samples were directly diluted in phosphate‐buffered saline and centrifuged for 10 minutes, at 3584 relative centrifugal force/G‐force (g) and +4°C. The obtained supernatants were divided into aliquots of 200 to 500 µL, each, and conserved in −80°C. Using appropriate transport conditions, the supernatants were transported to Innsbruck Medical University, Austria, where they were processed for molecular biology techniques.

#### 2.3.1 Nucleic acids extraction

The total nucleic acids extraction was executed using the QIAsymphony Sp automate (Cat No./ID: 9001297; Qiagen, Hilden, Germany) and the QIAsymphony DSP virus/Pathogen Mini Kit (Qiagen, Vienna, Austria) according to the manufacturer’s instructions. The extraction procedure was completed using 600 µL of original materials. A final extracted total nucleic acid of 150 µL was obtained.

#### 2.3.2 Screening of respiratory pathogens

The Fast Track DIAGNOSTICS (FTD) respiratory pathogens 21 Kit (FTD‐2‐64; Fast‐Track Diagnostics, Luxemburg S.à.r.l., 29, rue Henri Koch, L‐4354 Esch‐sur‐Alzette), a multiplex qRT‐PCR for the detection of pathogen genes by TaqMan Technology, was applied. PCR was accomplished as follow: 12.5 µL of PCR buffer, 1.5 µL of Master Mix (primer and probe sets one to five containing: Mix 1 for the detection of InfV‐A, InfV‐B, InfV‐A (H1N1) swl, and RV; Mix 2 for the screening of CoV‐NL63, CoV‐229E, CoV‐OC43, and CoV‐HKU1; Mix 3 for the identification of PIV‐2, PIV‐3, PIV‐4, and internal control; Mix 4 for the research of PIV‐1, MPV A/B, BoV, and *M. pneumoniae;* and Mix 5 for the detection of RSV A/B, AdV, EV, and PeV); and 1 µL of reverse transcriptase enzyme. PCR mixture was completed by 10 µL of extracted nucleic acids for each primer/probe set. Positive and negative controls were recommended. Thermocycling was achieved on a Rotor‐Gene 6000/Q (Qiagen, Hilden, Germany) using this program: reverse transcription reaction at 42°C for 15 minutes; then PCR reaction starting with initial denaturation at 94°C for 3 minutes followed by 40 cycles including a denaturation step at 94°C for 8 seconds; and an annealing and extension step at 60°C for 34 seconds.

#### 2.3.3 ***S. pneumoniae*** genome detection

*S. pneumoniae* genome detection was carried out by 16S ribosomal DNA (16S rDNA) qPCR using the Light Cycler Fast Start DNA Mas (#03003248001) kit as per the manufacturer's instructions. A 14 µL of Mastermix containing: MgCl_2_, 10× LC‐HYBR (a+b), forward primer (Strep‐F, 5’‐TGCAGAGCGTCCTTTGGTCTAT‐3,’ 50 µM), reverse primer (Strep‐R, 5’‐CTCTTACTCGTGGTTTCCAACTTGA‐3,’ 50 µM), probe (Strep‐TM, 5’‐ABI‐FAM‐TGGCGCCCATAAGCAACACTCGAA‐3’‐ABI‐Tamra, 20 µM), and H_2_O (Roche, Basel, Switzerland). The primers and probe were designed by Corless et al.^12^ Positive and negative controls were included. A final volume of a 20 µL PCR mixture was completed by 6 µL of extracted nucleic acids. PCR reaction was achieved in a Light Cycler 2.0 (Roche) with the following program: initial denaturation at 95°C for 7 minutes followed by a 50 cycles PCR, including a denaturation step at 95°C for 5 seconds, and an annealing and extension step at 62°C for 45 seconds. A final cooling step at 40°C over time was recommended. The PCR results were analyzed using the Light Cycler Software 4.05.

#### 2.3.4 Statistical analysis

The statistical calculations were evaluated using the Statistical Package for the Social Sciences (SPSS, version 20.0) with the assistance of statisticians from the Department of Epidemiology and Medical Statistics in FH‐UH of Sousse. The continuous variables (age of patients and the seasonal distribution of hospitalizations) were summarized using the mean ± standard deviation or median, interquartile range (IQR). The categorical variables (gender, age groups, seasonality of infection, and pathogenic infection rates) were described using proportions and percentages. A subgroup analysis was carried out to determine between the groups *P* values and on the basis of the between groups *P* values, in almost all variables, a pairwise comparison for the subgroup pairs was conducted. No missing data were revealed for the study population. The statistical calculations for the categorical variables were compared using Pearson’s chi‐square (*X* ^2^) or Fisher’s exact tests, as appropriate. The Fisher’s exact test was applied where cell counts below 5 were encountered. The odds ratio (OR) and 95% confidence intervals were estimated. All ORs are univariate. The binary logistic regression model was applied when more than two categories were observed. The ordinal regression model was used to study the impact of single, double, and multiple viral infections on the patient’s demography. The level of significance was set at *P* < = 0.05. No statistics were calculated when the number of infected subjects for individual pathogens was below 30 (minimally relevant effect size).^13^

## 3 RESULTS

### 3.1 Characteristics of the study population

Among the 515 enrolled patients, 59.0% were hospitalized in the PP ward, and 41.0% were admitted to the NN ward (G1 age group). The patients hospitalized in the PP ward (G2 and G3 age groups) were divided as follows: 40.0% of cases belonged to the group G2, and 19.0% were included in the group G3. A male predominance was observed in both wards with a total of 62.9% male cases (sex‐ratio = 1.69). The hospitalization due to ARIs occurred more in winter seasons with 31.0% in the period between September and December 2013 and 46.2% in the period of January‐March 2014 (Table 1).

### 3.2 Characteristics of severe respiratory disease

#### 3.2.1 Overview of the pathogenic infectious agents

*M. pneumoniae* was the only bacterial agent included in the multiplex qRT‐PCR of the FTD respiratory pathogens 21 kit. Not a single positive *M. pneumoniae* infection was found, and a total of 884 viruses were detected. The rate of positive tested individuals was 87.2% (449/515 specimens) versus 12.8% tested negative (66/515). More than the half of the tested samples were positive for RV (51.8%, 267/515), followed by RSV A/B (34.4%, 177/515). The *Coronavirus* group (CoVs), including CoV‐229E, CoV‐OC43, CoV‐NL63, and CoV‐HKU1, was detected in 18.4% of patients (95/515) in which the CoV‐229E was predominant (8.1%, 42/515) and was followed by CoV‐HKU1 (7.6%, 39/515). AdV was positive in 17.9% of tested samples (92/515) followed by *Parainfluenza virus* 1‐4 (PIVs, 10.9%, 56/515). PIV‐3 was the most frequently detected within PIVs (6.6%, 34/515). With a frequency of infection below 10.0%, MPV A/B (8.7%, 45/515); BoV (7.2%, 37/515); PeV (7.0%, 36/515), and EV (5.8%, 30/515) were detected. The influenza virus group (InfVs), including InfV‐A, InfV‐B, and InfV‐A (H1N1) swl, was occasionally positive (1.8%, 9/515). The uniplex qPCR for the 16S rDNA detected *S. pneumoniae* in 30.9% of the samples (159/515), (Table 2).

**Table 2:** Overview about the distribution of the total tested pathogenic infectious agents

Pathogenic agents	Positive infection	Single infection	Coinfection	Multiple infection
	No. (%)	No. (%)	No. (%)	No. (%)
RV	267 (51.8)	92 (17.9)	105 (20.4)	70 (13.6)
RSV A/B	177 (34.4)	55 (10.7)	73 (14.2)	49 (9.5)
*S. pneumoniae*	159 (30.9)	12 (2.3)	57 (11.1)	90 (17.5)
CoVs	95 (18.4)	13 (2.5)	25 (4.9)	57 (11.1)
CoV‐NL63	6 (1.2)	1 (0.2)	1 (0.2)	4 (0.8)
CoV‐229E	42 (8.2)	5 (1.0)	14 (2.7)	23 (4.5)
CoV‐OC43	8 (1.6)	2 (0.4)	0 (0.0)	6 (1.2)
CoV‐HKU1	39 (7.6)	5 (1.0)	10 (1.9)	24 (4.7)
AdV	92 (17.9)	7 (1.4)	46 (8.9)	39 (7.6)
PIVs	56 (10.9)	14 (2.7)	15 (2.9)	27 (5.2)
PIV‐1	6 (1.2)	3 (0.6)	2 (0.4)	1 (0.2)
PIV‐2	4 (0.8)	1 (0.2)	1 (0.2)	2 (0.4)
PIV‐3	34 (6.6)	10 (1.9)	6 (1.2)	18 (3.5)
PIV‐4	12 (2.3)	0 (0.0)	6 (1.2)	6 (1.2)
MPV A/B	45 (8.7)	2 (0.4)	23 (4.5)	20 (3.9)
BoV	37 (7.2)	4 (0.8)	12 (2.3)	21 (4.1)
PeV	36 (7.0)	0 (0.0)	6 (1.2)	30 (5.8)
EV	30 (5.8)	3 (0.6)	15 (2.9)	12 (2.3)
InfVs	9 (1.7)	1 (0.2)	4 (0.8)	4 (0.8)
InfV‐A	3 (0.6)	1 (0.2)	1 (0.2)	1 (0.2)
InfV‐B	1 (0.2)	0 (0.0)	1 (0.2)	0 (0.0)
H1N1 swl	5 (1.0)	0 (0.0)	2 (0.4)	3 (0.6)
*M. pneumoniae*	0 (0.0)	0 (0.0)	0 (0.0)	0 (0.0)

Abbreviations: AdV, adenovirus; BoV, bocavirus; CoVs, coronavirus group, CoV‐HKU1: coronavirus HKU1; CoV‐NL63, coronavirus NL63; CoV‐OC43, coronavirus OC43; CoV‐229E, coronavirus 229E; EV, enterovirus genus; H1N1, influenza virus A (H1N1) swl; InfVs, influenza virus group; InfV‐A, influenza virus A; InfV‐B, influenza virus B; *M. pneumoniae*: *Mycoplasma pneumoniae*; MPV A/B, metapneumovirus A/B; No., number; PeV, parechovirus genus; PIVs, parainfluenza virus group; PIV 1‐4, parainfluenza viruses 1‐4; RV, rhinovirus; RSV A/B, respiratory syncytial virus A/B; *S. pneumoniae*: *Streptococcus pneumoniae;* (%), percentage.

The pathogenic infection rates including single, double and multiple infections were estimated by dividing the number of positive samples detected for each pathogen by the total number of patients enrolled in the current study (515 subjects).

#### 3.2.2 Age and gender characteristics of the tested pathogens

The distribution of the pathogenic infectious agents, including viruses and *S. pneumoniae*, by age and gender is described in Tables 3 and 4 and Supporting Information Table S1. Viral infection was higher in patients hospitalized in the PP ward (90.8%, 276/304 infants vs 82.0%, 173/211 patients admitted in the NN ward, *P* < 0.001, OR = 2.16). Viral infectious agents were more frequently detected within male patients with 88.0% (285/324 males) of infection versus 85.9% (164/191 females) of infected female subjects. Regarding the groups of infants, viral infection was predominant in the cases belonging to the G2 group (91.8% vs 90.3% of infected cases from the G3 group). No statistical significance was revealed between viral infection and patient's gender or age (*P* = 0.49 and *P* = 0.66, respectively). Supporting Information Table S1 describes the distribution of individual viral infection by gender and age: RSV A/B was the single virus found to be statistically associated with gender (38.0% RSV A/B male cases vs 28.3% RSV A/B female cases, *P* = 0.02) and MPV A/B, BoV, as well as AdV infections, increased with the increasing age of patients (more frequent within the G3 group, *P* < 0.001). Table 4 describes the distribution of *S. pneumoniae* infections according to the demographic data of the infected subjects. *S. pneumoniae* was found to be more frequent in infants (39.1% vs 19.0% of neonates, *P* < 0.001, OR = 2.75) and male cases (32.4% vs 28.3% female cases, *P* = 0.32, OR = 0.82). *S. pneumoniae* was found to be age‐dependent (*P* < 0.001): it was predominant in cases aged 6 to 12 months (41.8% vs 37.9% of cases aged 28 days to 6 months vs 19.0% of cases aged 0‐28 days).

**Table 3:** Demographic and seasonal characteristics of viral infection

Patient's data		Negative* No (%)	Positive No (%)	*P* value^**^	OR [95% CI]^c^
Gender^a^	Male*	39 (12.0)	285 (88.0)	0.49	1
	Female	27 (14.1)	164 (85.9)		0.83 [0.49‐1.40]
Population category (ward of hospitalization)^a^	Neonates^*^	38 (18.0)	173 (82.0)	**<0.001**	**1**
	Infants	28 (9.2)	276 (90.8)		**2.16 [1.28‐3.65]**
Infants^a^	G2 (28 d‐ 6 m)^*^	20 (9.7)	186 (90.3)	0.66	1
	G3 (6‐12 m)	8 (8.2)	90 (91.8)		1.21 [0.51‐2.85]
Season^b^	Sep‐Dec 2013^*^	29 (18.1)	131 (81.9)	0.09	1
	Jan‐Mar 2014	27 (11.3)	211 (88.7)		1.73 [0.98‐3.05]
	Apr‐Jun 2014	4 (6.2)	61 (93.8)		3.37 [1.13‐10.02]
	Jul‐Sep 2014	3 (16.7)	15 (83.3)		1.10 [0.30‐4.07]
	Oct‐Dec 2014	3 (8.8)	31 (91.2)		2.28 [0.65‐7.99]
Total		66 (12.8)	449 (87.2)	**–**	**–**

Abbreviations: G2, age group 2; G3, age group 3.

The statistical associations between viral infection and the patient’s demographic data as well as the seasonal distribution of ARIs were established. The statistical calculations were performed on the total study population, including neonates and infants. The third and fourth rows of the table describe the distribution of viral infection between neonates and infants and then between the age groups of infants (age groups 2 and 3).

^a^ For parameters with two categories, the Chi‐square test (X^2^) was used to calculate the *P* value. The odds ratio (OR) and the associated 95% confidence interval [95% CI] were estimated. All ORs are univariate.

^b^ For variables with more than two categories (season), the binary logistic regression model was applied, calculating the *P* value, OR, and [95% CI].

^c^ The OR evaluates the association between the exposure of two properties and their outcome. The [95% CI] indicates the degree of uncertainty associated with the OR. All ORs are univariate.

^*^ Indicates the reference group used for the statistical calculations.

^**^ A value of *P* < = 0.05 was considered as significant and represented in bold.

**Table 4:** Socio‐demographic and seasonal characteristics of *S. pneumoniae* infection and its association with viral respiratory infection

*S. pneumoniae* infection		Negative^*^ No. (%)	Positive^a^ No. (%)	*P* value^**^	OR [95% CI]^b^
Socio‐demographic data					
Study population	Neonates^*^	171 (81.0)	40 (19.0)	**<0.001**	**1**
	Infants	185 (60.9)	119 (39.1)		**2.75 [1.81‐4.16]**
Infants (age groups)	G2 (28d–6 m)^*^	128 (62.1)	78 (37.9)	0.50	1
	G3 (6‐12 m)	57 (58.2)	41 (41.8)		1.18 [0.72‐1.92]
Gender	Male^*^	219 (67.6)	105 (32.4)	0.32	1
	Female	137 (71.7)	54 (28.3)		0.82 [0.55‐1.21]
Seasonal distribution	Sep‐Dec 2013^*^	93 (58.1)	67 (41.9)	**<0.001**	**1**
	Jan‐Mar 2014	151 (63.4)	87 (36.6)		**0.80 [0.53‐1.20]**
	Apr‐Jun 2014	61 (93.8)	4 (6.2)		**0.09 [0.03‐0.26]**
	Jul‐Sep 2014^c^	18 (100.0)	0		**–**
	Oct‐Dec 2014	33 (97.1)	1 (2.9)		**0.04 [0.00‐0.31]**
Viral infection					
Total	Negative^*^	54 (81.8)	12 (18.2)	**0.01**	**1**
	Positive	302 (67.3)	147 (32.7)		**2.19 [1.13‐4.22]**
Single	No^*^	56 (82.4)	12 (17.6)	0.051	1
	Yes	134 (70.2)	57 (29.8)		1.98 [0.98‐3.98]
Double	No^*^	53 (81.5)	12 (18.5)	**0.02**	**1**
	Yes	108 (66.7)	54 (33.3)		**2.20 [1.08‐4.47]**
Multiple	No^*^	58 (81.7)	13 (18.3)	**<0.001**	**1**
	Yes	60 (62.5)	36 (37.5)		**2.67 [1.29‐5.55]**

Abbreviations: G2: age group 2, G3: age group 3; *S. pneumoniae*: *Streptococcus pneumoniae*.

The statistical calculations were performed on the total study population, including neonates and infants. The third and fourth rows of the table describe the distribution of *S. pneumoniae* infection between neonates and infants and then between the age groups of infants (age groups 2 and 3).

^a^ S*. pneumoniae* infection rates were calculated as a fraction of the total cases defined by each category within the sociodemographic data and viral infection.

^b^ The odds ratio (OR) and the 95% confidence interval [95% CI] were established using the Chi‐square test (*X* ^2^) (or the Fisher's exact test) for variables with two categories, and the binary logistic regression model for variables with >2 categories. All ORs are univariate.

^c^ The OR for the category “Jul‐Sep 2014” of the variable “season” was left blank as a value of zero was obtained.

^*^ Describes the reference groups used for the statistical calculations.

^**^ *P* value was calculated using the *X* ^2^ test or the Fisher's exact test on SPSS. A value of *P* < = 0.05 was considered as significant and is represented in bold.

#### 3.2.3 Seasonal distribution of respiratory agents

The seasonal distribution of respiratory viral infection between 2013 and 2014 was variable and predominated the winter seasons: 131 infections were detected in September‐December 2013, 211 infected cases in the period of January‐March 2014, 61 infections in April‐June 2014, 15 infected cases in July‐September 2014, and 31 infections in October‐December 2014 (Table 3).

**Figure 1:**
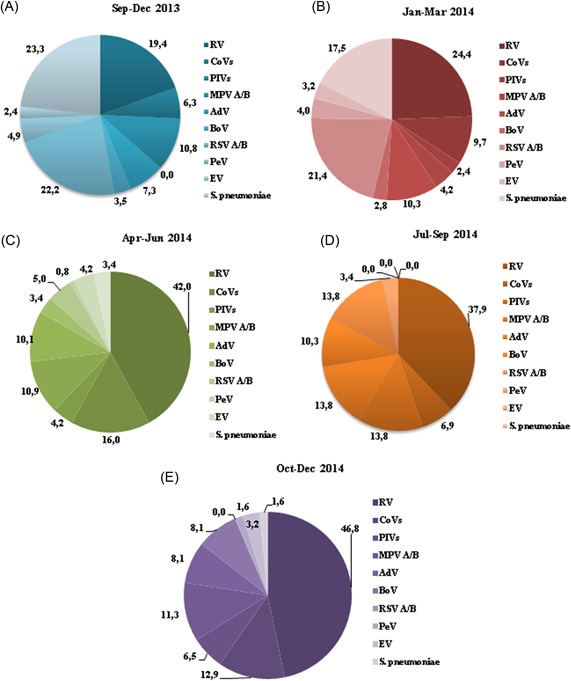
Seasonal distribution of the total tested pathogens between September 31, 2013 and December 31, 2014. The percentages were calculated as a fraction of the total pathogens detected in each season: 288 pathogens in the period of September‐December 2013 (A), 496 in January‐March 2014 (B), 119 in April‐June 2014 (C), 29 in July‐September 2014 (D), and 62 in October‐December 2014 (E). *Influenza viruses* were not included in this figure. Not a single *M.pneumoniae* infection was found. The *Coronavirus* and *Parainfluenza virus* types were grouped in CoVs and PIVs groups, respectively. AdV, adenovirus; BoV, bocavirus; CoVs, coronavirus group; EV, enterovirus genus; MPV A/B, metapneumovirus A/B; PeV, parechovirus genus; PIVs, parainfluenza virus group; RV, rhinovirus; RSV A/B, respiratory syncytial virus A/B; S. pneumoniae, *Streptococcus pneumoniae*

The seasonality of individual viral infection is summarized in Figure 1 and Supporting Information Table S2. Different patterns of viral circulation could be observed. In general, individual viral infection accounted for a larger proportion of ARIs in spring and winter. RV, RSV A/B, MPV A/B, CoVs, and PIVs infections were found to be significantly associated with season (*P* < 0.001). Viral infection was also observed in summer (July‐September 2014). Regarding bacteria, *S. pneumoniae* infection was found to be dependent on season (*P* < 0.001): the highest infection rates were observed in September‐December 2013 with 41.9% of infection, followed by 36.6% in January‐March 2014, 6.2% in April‐June 2014, and 2.9% in October‐December 2014. Not a single *S. pneumoniae* positivity was detected in the period of July‐September 2014 (Table 4).

### 3.3 Single and mixed pathogenic infections

Within subjects tested for viral infection, 37.0% were single positive, 31.4% were double infections, and 18.6% were multiple infections (3‐6 detected viruses per samples).

#### 3.3.1 Viral/bacterial coinfections

Table 2 and Supporting Information Table S3 reported the possible co‐occurrences of the tested pathogens for each virus/bacterial combination. Viral coinfections were detected mostly between RV and the following pathogens: RSV A/B (71 samples), CoVs (50 double infections), and AdV (45 coinfections). Other viral combinations were also found as follows: 31 RV‐MPV A/B double infections, 26 RV‐PIVs coinfected samples, 22 RV‐BoV, and RV‐PeV coinfections, each. Considering RSV A/B, double infections were detected with CoVs (34 coinfected samples), AdV (28 double infections), and PeV with 27 coinfected samples.

Regarding bacterial infections, a total of 147 samples (32.7%) were infected, in addition to *S. pneumoniae*, by at least one respiratory virus. Among these 147 positive samples, 57 were *S. pneumoniae* virus coinfections and 90 were multiple infected. *S. pneumoniae* viral combinations were described mostly by RSV A/B (44.5% of total *S. pneumoniae* infections) and RV (31.8%). The statistical association between *S. pneumoniae* and viral ARIs in tested subjects is described in Table 4. *S. pneumoniae* infection increases with viral infection (*P* = 0.01, OR = 2.19).

#### 3.3.2 Single and multiple infections

Among 12/66 of the negative viral infections (18.2%), *S. pneumoniae* was the single detected pathogen (Table 4). The possible association between single/double/multiple viral infections and demographic characteristics, seasonal distribution, as well as the individual viral infection is described in Table 5. Male patients were more frequently detected within multiple infections (69.5% of multiple infections). Infected female subjects were more found in single infections (40.8% of single infections). Single infections dominated the neonatal environment (53.9%). Double and multiple infections were more detected in the pediatric environment (71.3% and 76.8% of infection, respectively). The number of viruses detected per sample increases with the increasing age of patients (*P* < 0.001). For almost all pathogens, infection rates showed an increasing pattern within multiple infections. The exception was PIV‐1: its infection decreased with the increasing number of viruses detected per sample.

**Table 5:** Impact of single, double, and multiple viral infections on the patient's sociodemographic information

Socio‐demographic data		Single infection			Double infection			Multiple infection			Ordinal regression
**Infection / Statistics**		**No. (%)**	***P* value** ^**^	**OR [95% CI]**	**No. (%)**	***P* value** ^**^	**OR [95% CI]**	**No. (%)**	***P* value** ^**^	**OR [95% CI]**	***P* value** ^***^
Gender	Male^*^	**113 (59.2)**	0.87	1	**106 (65.4)**	0.32	1	**66 (68.8)**	0.06	1	0.07
	Female	**78 (40.8)**		1.05 [0.6‐1.8]	**56 (34.6)**		0.74 [0.4‐1.3]	**30 (31.2)**		0.55 [0.29‐1.04]	
Population	Neonates^*^	**103 (53.9)**	0.88	1	**48 (29.6)**	**<0.001**	**1**	**22 (22.9)**	**<0.001**	**1**	**<0.001**
	Infants	**88 (46.1)**		1.1 [0.6‐1.9]	**114 (70.4)**		**3.34 [1.8‐6.1]**	**74 (77.1)**		**5.1 [2.63‐10.1]**	
Infants	G2 (28d‐6 m)^*^	**63 (71.6)**	0.87	1	**75 (65.8)**	0.41	1	**48 (64.9)**	0.32	1	0.40
	G3 (6‐12 m)	**25 (28.4)**		0.92 [0.3‐2.2]	**39 (34.2)**		1.48 [0.6‐3.8]	**26 (35.1)**		**1.62 [0.61‐4.32]**	
Season	Sep‐Dec 2013^*^	**70 (36.6)**	0.68	1	**37 (22.8)**	**<0.01**	**1**	**24 (25.0)**	0.054	1	0.11
	Jan‐Mar 2014	**81 (42.4)**		0.80 [0.2‐3.2]	**82 (50.6)**		**0.17 [0.3‐0.8]**	**48 (50.0)**		0.20 [0.04‐1.1]	
	Apr‐Jun 2014	**23 (12.0)**		0.93 [0.2‐3.7]	**26 (16.0)**		**0.40 [0.08‐1.8]**	**12 (12.5)**		0.49 [0.09‐2.5]	
	Jul‐Sep 2014	**8 (4.2)**		1.9 [0.3‐10.3]	**2 (1.2)**		**0.86 [0.14‐5.3]**	**5 (5.2)**		0.68 [0.1‐4.5]	
	Oct‐Dec 2014	**9 (4.7)**		0.8 [0.1‐5.7]	**15 (9.4)**		**0.09 [0.009‐0.9]**	**7 (7.3)**		0.47 [0.05‐3.9]	
**Viral infection**		**No. (%)**	***P* value**	**OR [95% CI]**	**No. (%)**	***P* value**	**OR [95% CI]**	**No. (%)**	***P* value**	**OR [95% CI]**	***P* value** ^**^
RV	No	99 (51.8)	**<0.001**	**1**	57 (35.2)	**<0.001**	1	26 (27.1)	**<0.001**	**1**	**<0.001**
	Yes^*^	**92 (48.2)**		**30.7 [7.3‐128.8]**	**105 (64.8)**		**0.46 [0.3‐0.5]**	**70 (72.9)**		**29.1 [11.2‐75.3]**	
CoVs	No	178 (93.2)	0.12	1	137 (85.2)	**<0.01**	**1**	44 (45.8)	**<0.001**	**1**	**<0.001**
	Yes^*^	**13 (6.8)**		4.9 [0.6‐38.1]	**25 (14.8)**		**0.68 [0.61‐0.74]**	**52 (54.2)**		**82.7 [11.03‐620.1]**	
NL63^b^	No	190 (99.5)	–	–	161 (99.4)	–	–	92 (95.8)	–	–	N/A
	Yes	**1 (0.5)**		**–**	**1 (0.6)**		–	**4 (4.2)**		–	
229E	No	186 (97.4)	–	–	148 (91.4)	**<0.01**	1	73 (76.0)	**<0.001**	**1**	**<0.001**
	Yes^*^	**5 (2.6)**		**–**	**14 (8.6)**		**0.69 [0.63‐0.76]**	**23 (24.0)**		**0.50 [0.43‐0.59]**	
OC43^b^	No	189 (99.0)	–	–	162 (100.0)	–	–	90 (93.8)	–	–	N/A
	Yes	**2 (1.0)**		–	**0 (0.0)**		–	**6 (6.3)**		–	
HKU1	No	186 (97.4)	–	–	152 (93.8)	0.06	1	72 (75.0)	**<0.001**	**1**	**<0.001**
	Yes^*^	**5 (2.6)**		–	**10 (6.2)**		0.7 [0.6‐0.76]	**24 (25.0)**		**23.3 [3.1‐177.2]**	
PIVs	No	177 (92.7)	**0.02**	1	147 (90.7)	**<0.01**	**1**	71 (74.0)	**<0.001**	**1**	**<0.001**
	Yes^*^	**14 (7.3)**		**0.72 [0.6‐0.7]**	**15 (9.3)**		**0.69 [0.63‐0.75]**	**25 (26.0)**		**0.5 [0.42‐0.59]**	
PIV‐1^a^	No	188 (98.4)	–	–	160 (98.8)	–	–	95 (99.0)	–	–	N/A
	Yes	**3 (1.6)**		–	**2 (1.2)**		–	**1 (1.0)**		–	
PIV‐2^a^	No	190 (99.5)	–	–	161 (99.4)	–	–	94 (97.9)	–	–	N/A
	Yes	**1 (0.5)**		–	**1 (0.6)**		–	**2 (2.1)**		–	
PIV‐3	No	181 (94.8)	**0.05**	**1**	156 (96.3)	–	–	78 (81.2)	**<0.001**	**1**	**<0.001**
	Yes^*^	**10 (5.2)**		**0.72 [0.6‐0.7]**	**6 (3.7)**		–	**18 (18.8)**		**0.52 [0.45‐0.6]**	
PIV‐4^a^	No	191 (100.0)	–	–	156 (96.3)	–	–	90 (93.7)	–	–	N/A
	Yes	**0 (0.0)**		**–**	**6 (3.7)**		–	**6 (6.3)**		–	
MPV A/B	No	189 (99.0)	–	–	139 (85.8)	**<0.01**	**1**	76 (79.2)	**<0.001**	**1**	**<0.001**
	Yes^*^	**2 (1.0)**		**–**	**23 (14.2)**		**0.68 [0.62‐0.75]**	**20 (20.8)**		**0.51 [0.44‐0.60]**	
AdV	No	184 (96.3)	–	–	116 (71.6)	**<0.001**	**1**	57 (59.4)	**<0.001**	**1**	**<0.001**
	Yes^*^	**7 (3.7)**		**–**	**46 (28.4)**		**0.64 [0.57‐0.7]**	**39 (40.6)**		**47.9 [6.38‐359.4]**	
BoV	No	187 (97.9)	–	–	150 (92.6)	**0.02**	**1**	75 (78.1)	**<0.001**	**1**	**<0.001**
	Yes^*^	**4 (2.1)**		**–**	**12 (7.4)**		**0.69 [0.6‐0.7]**	**21 (21.9)**		**0.51 [0.43‐0.60]**	
RSV A/B	No	136 (71.2)	**<0.001**	**1**	89 (54.9)	**<0.001**	1	47 (49.0)	**<0.001**	**1**	**<0.001**
	Yes^*^	**55 (28.8)**		**0.66 [0.6‐0.07]**	**73 (45.1)**		**0.58 [0.50‐0.66]**	**49 (51.0)**		**72.9 [9.7‐546.8]**	
PeV	No	191 (100.0)	–	–	156 (96.3)	–	–	66 (68.8)	**<0.001**	**1**	**<0.001**
	Yes^*^	**0 (0.0)**		–	**6 (3.7)**		–	**30 (31.3)**		**0.48 [0.40‐0.57]**	
EV	No	188 (98.4)	–	–	147 (90.7)	**<0.01**	**1**	84 (87.5)	**<0.01**	**1**	**<0.001**
	Yes^*^	**3 (1.6)**		–	**15 (9.3)**		**0.69 [0.63‐0.75]**	**12 (12.5)**		**0.54 [0.46‐0.62]**	
InfVs^a^	No	190 (99.5)	–	–	158 (97.5)	–	–	92 (95.8)	–	–	N/A
	Yes	**1 (0.5)**		–	**4 (2.5)**		–	**4 (4.2)**		–	
InfV‐A^a^	No	190 (99.5)	–	–	161 (99.4)	–	–	95 (99.0)	–	–	N/A
	Yes	**1 (0.5)**		–	**1 (0.6)**		–	**1 (1.0)**		–	
InfV‐B^a^	No	191 (100.0)	–	–	161 (99.4)	–	–	96 (100.0)	–	–	N/A
	Yes	**0 (0.0)**		–	**1 (0.6)**		–	**0 (0.0)**		–	
H1N1^a^	No	191 (100.0)	–	–	160 (98.8)	–	–	93 (96.9)	–	–	N/A
	Yes	**0 (0.0)**		–	**2 (1.2)**		–	**3 (3.1)**		–	
Total		**191 (37.0)**	–	–	**162 (31.4)**	–	–	**96 (18.6)**	–	–	–

Abbreviations: AdV, adenovirus; BoV, bocavirus; CoVs, coronavirus group; EV, enterovirus genus; G2, age group 2; G3, age group 3; HKU1, coronavirus HKU1; H1N1, influenza virus A (H1N1) swl; InfVs, influenza virus group; InfV‐A, influenza virus A; InfV‐B, influenza virus B; MPV A/B, metapneumovirus A/B; NL63, coronavirus NL63; No., number; OC43, coronavirus OC43; PeV, parechovirus genus; PIVs, parainfluenza virus group; PIV 1‐4, parainfluenza viruses 1‐4; RSV A/B, respiratory syncytial virus A/B; RV, rhinovirus; (%): percentage; 229E, coronavirus 229E.

The percentages were calculated by dividing the number of cases defined by each row by the total number of infections in each column (191 single infections, 162 double infections, and 96 multiple infections). The numbers and percentages of positive infections detected for each virus (Yes) and described in single, double, and multiple infections are represented in bold.

The statistical associations comparing individual virus and single/double/multiple infections were calculated.

^a^ The statistical calculations comparing individual viral infection and single/double/multiple infections were not established for viruses with a low number of infected samples because the low number of infections will make the associations ambiguous. N/A: not applicable statistical test.

^*^ Represents the reference group used for the statistical calculations.

^**^ *P* values were calculated using the Chi‐square test (*X* ^2^) or Fisher's exact test on SPSS. The odds‐ratio (OR) and the 95% confidence interval [95% CI] were established. For the variables with >2 categories (season), the binary logistic regression model was applied to determine the OR and [95% CI]. All ORs are univariate.

^***^ *P* value was estimated using the ordinal regression model on SPSS, which allows comparing between single, double, and multiple viral infections.

*P* ≦ 0.05 were considered as significant and are represented in bold with the OR and [95% CI].

## 4 DISCUSSION

This study investigates the etiology of ARIs in hospitalized neonates and infants under 1 year of age in the area of Sousse, Tunisia, and associates viral/bacterial infection with their seasonal distribution and with the sociodemographic data of the enrolled patients.

The multiplex qRT‐PCR detected a high rate of viral infection, estimated at 87.2%. Comparable studies have shown variable detection rates ranging from less than 50.0% to more than 85.0%.^14^, ^15^, ^16^ The wide differences observed in the detection rates of infectious pathogenic agents could be interpreted by the heterogeneity of the study population, the genetic variability between populations, the nature of biological material used for the detection of respiratory pathogens by multiplex assays, the technique itself, and the climate/regional factors that influence the appearance of respiratory agents.^16^, ^17^

In this study, RV and RSV A/B were the most frequently detected viruses and were found in 51.8% and 34.4% of tested specimens, respectively. Both are known as the crucial causes of ARIs in children.^18^, ^19^ In addition, a male predominance was observed within infected subjects for all respiratory viruses in which RSV A/B was the single pathogen found to be significantly associated with gender (*P* = 0.02). The hypothesis that RSV A/B and other detected viruses were predominant within male subjects remains unclear. This finding could be explained by the genetic factors described in both genders.^20^

Despite the fact that RSV A/B is frequent worldwide and its distribution is consistent with a variety of reports in both developing and developed countries, RV has been increasingly reported by previous studies^21^, ^22^, ^23^ and was the most frequently detected pathogen in the present study. Its high prevalence makes its infection more serious. Although RV is described as one of the most commonly identified viruses in persons experiencing acute respiratory illnesses, RV has not been fully appreciated as a pathogen causing severe clinical symptoms. Besides, RV is known to play an important role as the cause of asthma.^24^ Thus, the implementation of RV should be considered as a standard assay in routine laboratory testing in low and middle‐income countries. However, the qRT‐PCR techniques are still expensive and cannot be generalized and routinely applied in these countries. Still, it should be considered to introduce these techniques in some public hospitals to provide information on which rapid tests should be considered. Before that, it will be necessary to perform further studies evaluating the cost/benefit before initiating these tests in routine.

Children with RV and RSV A/B infections are also exposed to a variety of other respiratory viruses. Interestingly, in the present study, 31.4% double viral infections and 18.6% infections by 3 to 6 viruses were observed. RV‐RSV A/B was the most frequently detected combination and was found in 71 of the 515 tested samples. Many studies have revealed that an important number of pediatric patients with ARIs become simultaneously infected with multiple respiratory viruses, but few studies have focused on analyzing viral coinfections. It has been estimated that the effect of mixed viral infections on a patient's health may depend on which viruses coinfect.^24^, ^25^

In the present study, the association between viral co/multiple infections and the demographic distribution of patients as well as the seasonality of detected pathogens was established. The findings show that viral co/multiple infections occur mainly in winter seasons (October‐December 2013 and January‐March 2014) and are predominant in cases aged 28 days to 6 months (vs neonates [0‐28 days] and infants aged 6‐12 months, *P* < 0.001). Nonetheless, no significant association was revealed with gender or season.

Since the discovery of novel respiratory agents, the CoVs group was reported in 18.6% of the enrolled cases, which ranks the third most frequently identified virus after RV and RSV A/B. In contrast, several studies report that CoVs ARIs remain rare and are not implemented in the routine laboratory testing.^26^ A single Tunisian research carried out over 3 consecutive years (2009‐2011) has found that CoV was rare and mainly observed in 2010‐2011 with a total of 5/160 positive infections. No description of the distribution of CoV subtypes was performed.^11^ The present study describes for the first time in Tunisia the epidemiology of the most known CoVs subtypes: CoV‐2299E, CoV‐OC43, CoV‐NL63, and CoV‐HKU1, in which CoV‐229E was found to be the most dominated. If the discrepancy may be due to the different tests used in the studies or the appearance of CoV as a more frequent pathogen found in Tunisia remains to be investigated.

Furthermore to CoVs, ARI due to AdV was frequent in this study and its total positive rate was 17.8% in which 77.1% of its infections were in infants. Most of the AdV infections were detected in combination with other viruses (46/92 AdV infections). This observation is in contrast with the reports described by Huang et al in 2011 and Liu et al in 2015, in which AdV was more detected in single infections.^27^, ^28^ Because there are no prospective and because of the large randomized controlled trials of AdV infections, the treatments of this pathogen are controversial.

PIV infection has been described in 10.8% of enrolled cases. In line with other publications, PIVs were reported in 10.0% to 11.5% of all acute respiratory hospitalizations, respectively.^29^, ^30^, ^31^ Likewise, this study found that PIV‐3 was the most detected virus within the PIV group. It has been described in the literature that the epidemics of PIV‐3 respiratory infection are responsible for considerable economic losses due to hospitalizations, medical costs, and mortality in children worldwide.^30^, ^31^

Additional pathogens were described in this study and accounted for less than 10.0% of the infections, including MPV A/B, BoV, PeV, EV, and InfVs group. MPV A/B was reported in 8.7% of specimens, in which 23/45 of them were coinfected with other respiratory viruses and only 2/45 were monoinfected. Dual infections between MPV A/B and RSV A/B have been reported widely in the literature. However, our finding shows that only seven MPV A/B‐RSV A/B coinfections were detected. This result is supported by Cebey‐Lopez et al’s^25^ study, in which a low proportion of monoinfection by MPV A/B was described.

MPV A/B was followed by BoV, indicating a 7.1% infection rate. Infection by BoV has recently attracted attention worldwide because the incidence and respiratory infection of this pathogen vary widely and often involve co/multiple infections with other potential pathogens,^32^, ^33^ which is in agreement with the present study.

Members of the *Picornaviridae* family, PeV, and EV, were screened. Although, PeV is frequently isolated from patients with respiratory infection and is thought to be associated with disease. The qRT‐PCR was shown as the best way to detect an EV or PeV, but still little is known about the prognosis of these viruses.^34^, ^35^ In the present study, it was of significance to describe the circulation of EV and PeV in ARIs in the area of Sousse. In 5.8% and 7% of the 515 enrolled subjects, EV and PeV were detected, respectively. The lowest prevalence of ARIs in this study has been attributed to the InfVs group with an infection rate by 1.8%. This finding was not cited by other studies in which InfVs ARIs were frequent.^26^ The rare presence of InfVs infection in the study population could be related to the age of patients in which all enrolled subjects were aged below 1 year.

As described above, *M. pneumoniae* was not detected in any specimen. The absence of *M. pneumoniae* mediated ARI in this study could be elucidated by the rare frequencies of M*. pneumoniae* infections in neonates and infants. A similar finding was reported by Halaji et al^36^ showing that *M. pneumoniae* ARI in children aged 1 month to 15 years is infrequent (7/150 positive samples).

In addition to the respiratory viruses/bacteria screened in this study, negative infections were found. In 66 samples (12.8%), the multiplex assay showed neither respiratory viruses nor *M. pneumonia* infection. However, within the 12/66 negative samples, the additionally performed uniplex qPCR for *S. pneumonia* appeared to be positive. Alternatively, the remaining negative samples (54/66) did not contain any pathogen that was screened for and the ARI may be not linked to infectious conditions. This may be due to the presence of pathogens not covered by the multiplex approach of this study. Bacteria genome detection by qPCR identified *S. pneumoniae* in 159 out of 515 tested samples (30.9%). The high prevalence of *S. pneumoniae* in children hospitalized with ARIs is supported by other studies, showing that pneumonia, acute cough, bronchitis, and lower ARIs are often caused by infections with viruses or *S. pneumoniae*.^37^ In addition, *S. pneumoniae* could be found to colonize the nasopharynx during RSV A/B infection in young children, which was associated with increased severity.^38^ In accordance, 82.6% of *S. pneumoniae* infections were coinfected with a respiratory virus in which RSV A/B was the most identified pathogen.

With the changes occurring in the demographic situation of patients, the environmental characteristics, the seasonality, and social habits throughout the world, the emergence of new respiratory pathogens still increases each year. This consequence makes clinicians more confused about which pathogen is responsible for ARIs, and thus preventions should be taken to minimize the risk of ARIs or the circulation of nosocomial infections in the community.

Thus, regardless of the above, the findings listed in the present study need to be interpreted in consideration with the limitations of this study. First, the data were collected only from a single study site (FH‐UH of Sousse). Second, this study was conducted for a short study period (September 2013‐December 2014). Due to the sampling size collected during this year, the wide range of tested pathogens, and the costs of materials and reagents, it was not possible to increase the sampling period. Although this study permits the detection of a wide range of pathogens, it is focused on the identification of viruses and neglects a wide range of other infectious pathogenic agents (viruses, bacteria, and fungi) responsible for the manifestation of severe respiratory diseases. Finally, it would be interesting to include the patient's clinical manifestations and their association with viral/bacterial infection. In contrast to data from older children, we had, unfortunately, no access to clinical manifestations of neonates.

## 5 CONCLUSION

In summary, the high prevalence of etiology associated to respiratory infections of relevant pathogens highlights the importance of sustaining national surveillance of ARIs to clearly estimate the role of associated pathogens and establish the burden of disease. The findings of this study address several important gaps in the literature. The CoVs and AdV ARIs were frequent in neonates and infants in Tunisia. The CoVs subtypes were described for the first time in Tunisia and revealed that CoV‐229E was the most frequently detected. In spite of the common knowledge that RSV A/B is the most common respiratory agent responsible for ARIs, RV was found to be more prevalent in this population, which highlights the interest of cost benefits studies concerning its implementation in the routine laboratory testing. Given the multiplicity of these findings, this study provides a useful starting point for a better understanding about the circulation of these pathogens in the area of Sousse and the seasonal/demographic attributable to viral/bacterial infection. It will contribute data relevant to the development of prevention strategies, which will help in clinical decisions and allow considering antiviral therapy in clinical routine. The availability of vaccines in Tunisia only against InfVs infections will afford ongoing vaccine research and development on many other leading viral pathogens in future.

## CONFLICTS OF INTEREST

The authors declare no conflicts of interest.

## FUNDING

This study was supported by the Ministry of Higher Education and Scientific Research in Tunisia and Innsbruck Medical University in Austria (the FWF, Horos doctoral Program, W1253‐B24).

## Supporting information


**Supporting information**
