# Supplementary material for: Demographic and seasonal characteristics of respiratory pathogens in neonates and infants aged 0 to 12 months in the Central‐East region of Tunisia
Source: J Med Virol. 2018 Nov 21;91(4):570–81. doi: 10.1002/jmv.25347 (PMC6492255; doi:10.1002/jmv.25347)
Supplement: Supplementary file 1 — Supporting information [file JMV-91-570-s001.docx]

**Table S1. Distribution of individual viral infection in neonates and infants aged <1 year according to gender and age groups. The statistical associations between individual virus and the demographic data of infected subjects were established.**

| **Gender/age** | | **Gender** | | **P-value^**^** | **Age groups** | | | **P-value^**^** |
| --- | --- | --- | --- | --- | --- | --- | --- | --- |
| **Viral agents** | | ***Male**** | ***Female*** |  | ***G1*(Neonates)*** | ***G2 (Infants)*** | ***G3 (Infants)*** |  |
| **RV** | ***N**** | 153 (47.2) | 95 (49.7) | 0.58 | 114 (54.0) | 93 (45.1) | 41 (41.8) | 0.07 |
|  | ***P*** | **171 (52.8)** | **96 (50.3)** |  | **97 (46.0)** | **113 (54.9)** | **57 (58.2)** |  |
| **CoVs** | ***N**** | 263 (81.2) | 157 (82.2) | 0.81 | 178 (84.4) | 168 (81.6) | 74 (75.5) | 0.07 |
|  | ***P*** | **61 (18.8)** | **34 (17.8)** |  | **33 (15.6)** | **38 (18.4)** | **24 (24.5)** |  |
| **NL63** | ***N*** | 320 (98.8) | 189 (99.0) | N/A | 210 (99.5) | 202 (98.1) | 97 (99.0) | N/A |
|  | ***P*** | **4 (1.2)** | **2 (1.0)** |  | **1 (0.5)** | **4 (1.9)** | **1 (1.0)** |  |
| **229E** | ***N**** | 300 (92.6) | 173 (90.6) | 0.41 | 202 (95.7) | 183 (88.8) | 88 (89.8) | **0.02** |
|  | ***P*** | **24 (7.4)** | **18 (9.4)** |  | **9 (4.3)** | **23 (11.2)** | **10 (10.2)** |  |
| **OC43** | ***N*** | 318 (98.1) | 189 (99.0) | N/A | 205 (97.2) | 205 (99.5) | 97 (99.0) | N/A |
|  | ***P*** | **6 (1.9)** | **2 (1.0)** |  | **6 (2.8)** | **1 (0.5)** | **1 (1.0)** |  |
| **HKU1** | ***N**** | 297 (91.7) | 179 (93.7) | 0.39 | 194 (91.9) | 196 (95.1) | 86 (87.8) | 0.07 |
|  | ***P*** | **27 (8.3)** | **12 (6.3)** |  | **17 (8.1)** | **10 (4.9)** | **12 (12.2)** |  |
| **PIVs** | ***N**** | 292 (90.1) | 167 (87.4) | 0.37 | 188 (89.1) | 188 (91.3) | 83 (84.7) | 0.20 |
|  | ***P*** | **32 (9.9)** | **24 (12.6)** |  | **23 (10.9)** | **18 (8.7)** | **15 (15.3)** |  |
| **PIV-1** | ***N*** | 321 (99.1) | 188 (98.4) | N/A | 207 (98.1) | 205 (99.5) | 97 (99.0) | N/A |
|  | ***P*** | **3 (0.9)** | **3 (1.6)** |  | **4 (1.9)** | **1 (0.5)** | **1 (1.0)** |  |
| **PIV-2** | ***N*** | 322 (99.4) | 189 (99.0) | N/A | 210 (99.5) | 204 (99.0) | 97 (99.0) | N/A |
|  | ***P*** | **2 (0.6)** | **2 (1.0)** |  | **1 (0.5)** | **2 (1.0)** | **1 (1.0)** |  |
| **PIV-3** | ***N**** | 305 (94.1) | 176 (92.1) | 0.38 | 194 (91.9) | 198 (96.1) | 89 (90.8) | 0.11 |
|  | ***P*** | **19 (5.9)** | **15 (7.9)** |  | **17 (8.1)** | **8 (3.9)** | **9 (9.2)** |  |
| **PIV-4** | ***N*** | 316 (97.5) | 187 (97.9) | N/A | 210 (99.5) | 199 (96.6) | 94 (95.9) | N/A |
|  | ***P*** | **8 (2.5)** | **4 (2.1)** |  | **1 (0.5)** | **7 (3.4)** | **4 (4.1)** |  |
| **MPV A/B** | ***N**** | 295 (91.0) | 175 (91.6) | 0.82 | 210 (99.5) | 178 (86.4) | 82 (83.7) | **<0.001** |
|  | ***P*** | **29 (9.0)** | **16 (8.4)** |  | **1 (0.5)** | **28 (13.6)** | **16 (16.3)** |  |
| **AdV** | ***N**** | 266 (82.1) | 157 (82.2) | 0.97 | 190 (90.0) | 161 (78.2) | 72 (73.5) | **<0.001** |
|  | ***P*** | **58 (17.9)** | **34 (17.8)** |  | **21 (10.0)** | **45 (21.8)** | **26 (26.5)** |  |
| **BoV** | ***N**** | 298 (92.0) | 180 (94.2) | 0.33 | 205 (97.2) | 187 (90.8) | 86 (87.8) | **<0.001** |
|  | ***P*** | **26 (8.0)** | **11 (5.8)** |  | **6 (2.8)** | **19 (9.2)** | **12 (12.2)** |  |
| **RSV A/B** | ***N**** | 201 (62.0) | 137 (71.7) | **0.02** | 140 (66.4) | 128 (62.1) | 70 (71.4) | 0.27 |
|  | ***P*** | **123 (38.0)** | **54 (28.3)** |  | **71 (33.6)** | **78 (37.9)** | **28 (28.6)** |  |
| **PeV** | ***N**** | 299 (92.3) | 180 (94.2) | 0.40 | 200 (94.8) | 185 (89.8) | 94 (95.9) | 0.06 |
|  | ***P*** | **25 (7.7)** | **11 (5.8)** |  | **11 (5.2)** | **21 (10.2)** | **4 (4.1)** |  |
| **EV** | ***N**** | 304 (93.8) | 181 (94.8) | 0.66 | 205 (97.2) | 189 (91.7) | 91 (92.9) | 0.051 |
|  | ***P*** | 20 (6.2) | 10 (5.2) |  | **6 (2.8)** | **17 (8.3)** | **7 (7.1)** |  |
| **InfVs** | ***N*** | 317 (97.8) | 189 (99.0) | N/A | 206 (97.6) | 203 (89.5) | 97 (99.0) | N/A |
|  | ***P*** | **7 (2.2)** | **2 (1.0)** |  | **5 (2.4)** | **3 (1.5)** | **1 (1.0)** |  |
| **InfV-A** | ***N*** | 321 (99.1) | 191 (100.0) | N/A | 211 (100.0) | 204 (99.0) | 97 (99.0) | N/A |
|  | ***P*** | **3 (0.9)** | **0** |  | **0 (0.0)** | **2 (1.0)** | **1 (1.0)** |  |
| **InfV-B** | ***N*** | 323 (99.7) | 191 (100.0) | N/A | 210 (99.5) | 206 (100.0) | 98 (100.0) | N/A |
|  | ***P*** | **1 (0.3)** | **0** |  | **1 (0.5)** | **0 (0.0)** | **0 (0.0)** |  |
| **H1N1** | ***N*** | 321 (99.1) | 189 (99.0) | N/A | 207 (98.1) | 205 (99.5) | 98 (100.0) | N/A |
|  | ***P*** | **3 (0.9)** | **2 (1.0)** |  | **4 (1.9)** | **1 (0.5)** | **0 (0.0)** |  |

The percentages were estimated dividing the number of infected/non infected cases on the total number of cases defined by each column. The number and rates of positive viral infection for each parameter (gender and age) are represented in bold.

* Indicates the reference groups used for the statistical associations.

^**^ P-value was estimated using the Chi-square (X^2^) test or the Fisher’s exact test on SPSS. No statistical calculations were evaluated for InfVs group, PIV-1, PIV-2, PIV-4, CoV-NL63, and CoV-OC43 because the less number of infected samples by each pathogen will make the comparisons invalid (not applicable: N/A). A value of p<=0.05 was considered as significant and represented in bold.

Abbreviations: N: negative viral infection, P: positive viral infection, G1: age group 1 (0-28 days), G2: age group 2 (28 days-6 months) , G3: age group 3 (6-12 months), RV: *Rhinovirus*, CoVs: *Coronavirus* group, NL63: *Coronavirus NL63*, 229E: *Coronavirus 229E*, OC43: *Coronavirus OC43*, HKU1: *Coronavirus HKU1*, PIVs: *Parainfluenza virus* group, PIV 1-4: *Parainfluenza viruses 1-4*, MPV A/B: *Metapneumovirus A/B*, AdV: *Adenovirus*, BoV: *Bocavirus*, RSV A/B: *Respiratory Syncytial virus A/B*, PeV: parechovirus genus, EV: enterovirus genus, InfVs: *Influenza virus* group, InfV-A: *Influenza virus A*, InfV-B: *Influenza virus B*, and H1N1: *Influenza virus A (H1N1) swl*.

**Table S2. Seasonal distribution of individual viral infection in patients hospitalized for ARIs in the area of Sousse, Tunisia in the period of September 31, 2013- December 31, 2014. The statistical significance between each virus and season was estimated.**

| **Season/viral agents** | | ***September-December 2013**** | ***January-March 2014*** | ***April-June 2014*** | ***July-September 2014*** | ***October-December 2014*** | **P-value^**^** |
| --- | --- | --- | --- | --- | --- | --- | --- |
| **RV** | ***N**** | 104 (65.0) | 117 (49.2) | 15 (23.1) | 7 (38.9) | 5 (14.7) | **<0.001** |
|  | ***P*** | **56 (35.0)** | **121 (50.8)** | **50 (76.9)** | **11 (61.1)** | **29 (85.3)** |  |
| **CoVs** | ***N**** | 142 (88.8) | 190 (79.8) | 46 (70.8) | 16 (88.9) | 26 (76.5) | **0.03** |
|  | ***P*** | 18 (11.2) | 48 (20.2) | 19 (29.2) | 2 (11.1) | 8 (23.5) |  |
| **NL63** | ***N*** | 157 (98.1) | 235 (98.7) | 65 (100.0) | 18 (100.0) | 34 (100.0) | N/A |
|  | ***P*** | **3 (1.9)** | **3 (1.3)** | **0** | **0** | **0** |  |
| **229E** | ***N**** | 156 (97.5) | 217 (91.2) | 57 (87.7) | 17 (94.4) | 26 (76.5) | **<0.001** |
|  | ***P*** | **4 (2.5)** | **21 (8.8)** | **8 (12.3)** | **1 (5.6)** | **8 (23.5)** |  |
| **OC43** | ***N*** | 159 (99.4) | 234 (98.3) | 62 (95.4) | 18 (100.0) | 34 (100.0) | N/A |
|  | ***P*** | **1 (0.6)** | **4 (1.7)** | **3 (4.6)** | **0** | **0** |  |
| **HKU1** | ***N**** | 150 (93.8) | 218 (91.6) | 57 (87.7) | 17 (94.4) | 34 (100.0) | 0.21 |
|  | ***P*** | **10 (6.3)** | **20 (8.4)** | **8 (12.3)** | **1 (5.6)** | **0** |  |
| **PIVs** | ***N**** | 129 (80.6) | 226 (95.0) | 60 (92.3) | 14 (77.8) | 30 (88.2) | **<0.001** |
|  | ***P*** | 31 (19.4) | 12 (5.0) | 5 (7.7) | 4 (22.2) | 4 (11.8) |  |
| **PIV-1** | ***N*** | 154 (96.3) | 238 (100.0) | 65 (100.0) | 18 (100.0) | 34 (100.0) | N/A |
|  | ***P*** | **6 (3.8)** | **0** | **0** | **0** | **0** |  |
| **PIV-2** | ***N*** | 160 (100.0) | 235 (98.7) | 65 (100.0) | 17 (94.4) | 34 (100.0) | N/A |
|  | ***P*** | **0** | **3 (1.3)** | **0** | **1 (5.6)** | **0** |  |
| **PIV-3** | ***N**** | 141 (88.1) | 231 (97.1) | 62 (95.4) | 15 (83.3) | 32 (94.1) | **<0.001** |
|  | ***P*** | **19 (11.9)** | **7 (2.9)** | **3 (4.6)** | **3 (16.7)** | **2 (5.9)** |  |
| **PIV-4** | ***N*** | 154 (96.3) | 236 (99.2) | 63 (96.9) | 18 (100.0) | 32 (94.1) | N/A |
|  | ***P*** | **6 (3.8)** | **2 (0.8)** | **2 (3.1)** | **0** | **2 (5.9)** |  |
| **MPV A/B** | ***N**** | 160 (100.0) | 217 (91.2) | 52 (80.0) | 14 (77.8) | 27 (79.4) | **<0.001** |
|  | ***P*** | **0** | **21 (8.8)** | **13 (20.0)** | **4 (22.2)** | **7 (20.6)** |  |
| **AdV** | ***N**** | 139 (86.9) | 187 (78.6) | 53 (81.5) | 15 (83.3) | 29 (85.3) | 0.31 |
|  | ***P*** | **21 (13.1)** | **51 (21.4)** | **12 (18.5)** | **3 (16.7)** | **5 (14.7)** |  |
| **BoV** | ***N**** | 150 (93.8) | 224 (94.1) | 61 (93.8) | 14 (77.8) | 29 (85.3) | 0.056 |
|  | ***P*** | **10 (6.2)** | **14 (5.9)** | **4 (6.2)** | **4 (22.2)** | **5 (14.7)** |  |
| **RSV A/B** | ***N**** | 96 (60.0) | 132 (55.5) | 59 (90.8) | 17 (94.4) | 34 (100.0) | **<0.001** |
|  | ***P*** | **64 (40.0)** | **106 (44.5)** | **6 (9.2)** | **1 (5.6)** | **0** |  |
| **PeV** | ***N**** | 146 (91.3) | 218 (91.6) | 64 (98.5) | 18 (100.0) | 33 (97.1) | 0.18 |
|  | ***P*** | **14 (8.8)** | **20 (8.4)** | **1 (1.5)** | **0** | **1 (2.9)** |  |
| **EV** | ***N**** | 153 (95.6) | 222 (93.3) | 60 (92.3) | 18 (100.0) | 32 (94.1) | 0.72 |
|  | ***P*** | **7 (4.4)** | **16 (6.7)** | **5 (7.7)** | **0** | **2 (5.9)** |  |
| **InfVs** | ***N*** | 158 (98.8) | 231 (97.1) | 65 (100.0) | 18 (100.0) | 34 (100.0) | N/A |
|  | ***P*** | **2 (1.2)** | **7 (2.9)** | **0** | **0** | **0** |  |
| **InfV-A** | ***N*** | 160 (100.0) | 235 (98.7) | 65 (100.0) | 18 (100.0) | 34 (100.0) | N/A |
|  | ***P*** | **0** | **3 (1.2)** | **0** | **0** | **0** |  |
| **InfV-B** | ***N*** | 159 (99.4) | 238 (100.0) | 65 (100.0) | 18 (100.0) | 34 (100.0) | N/A |
|  | ***P*** | **1 (0.6)** | **0** | **0** | **0** | **0** |  |
| **H1N1** | ***N*** | 159 (99.4) | 234 (98.3) | 65 (100.0) | 18 (100.0) | 34 (100.0) | N/A |
|  | ***P*** | **1 (0.6)** | **4 (1.7)** | **0** | **0** | **0** |  |

The percentages were estimated dividing the number of infected/non infected cases on the total number of cases defined by each column. The number and rates of positive viral infection for each pathogen are represented in bold.

^*^ Indicates the reference groups used for the statistical associations.

^**^ P-value was estimated using the Chi-square (X^2^) test or the Fisher’s exact test on SPSS. A value of p<=0.05 was considered as significant and represented in bold.

Abbreviations: N: negative, P: positive, RV: *Rhinovirus*, CoVs: *Coronavirus* group, NL63: *Coronavirus NL63*, 229E: *Coronavirus 229E*, OC43: *Coronavirus OC43*, HKU1: *Coronavirus HKU1*, PIVs: *Parainfluenza virus* group, PIV 1-4: *Parainfluenza viruses 1-4*, MPV A/B: *Metapneumovirus A/B*, AdV: *Adenovirus*, BoV: *Bocavirus*, RSV A/B: *Respiratory Syncytial virus A/B*, PeV: parechovirus genus, EV: enterovirus genus, InfVs: *Influenza virus* group, InfV-A: *Influenza virus A*, InfV-B: *Influenza virus B*, and H1N1: *Influenza virus A (H1N1) swl*.

**Table S3. Overview of the number of viral/bacterial co-infections detected between the total tested pathogenic agents. A maximum of 6 pathogens per sample were detected.**

| **Co-infections^a^** | | | | | | | | | | | | | | | | | | | | | |  |
| --- | --- | --- | --- | --- | --- | --- | --- | --- | --- | --- | --- | --- | --- | --- | --- | --- | --- | --- | --- | --- | --- | --- |
|  | **InfV-A** | **InfV-B** | **H1N1** | **InfVs** | **RV** | **NL63** | **229E** | **OC43** | **HKU1** | **CoVs** | **PIV-1** | **PIV-2** | **PIV-3** | **PIV-4** | **PIVs** | **MPV A/B** | **AdV** | **BoV** | **RSV A/B** | **PeV** | **EV** | ***S. pneumoniae*** |
| **InfV-A** |  | 0 | 0 | 3 | 1 | 0 | 0 | 0 | 1 | 1 | 0 | 0 | 0 | 0 | 0 | 0 | 1 | 0 | 0 | 0 | 0 | 0 |
| **InfV-B** | - |  | 0 | 1 | 0 | 0 | 0 | 0 | 0 | 0 | 0 | 0 | 0 | 0 | 0 | 0 | 0 | 0 | 1 | 0 | 0 | 0 |
| **H1N1** | - | - |  | 5 | 4 | 0 | 0 | 1 | 0 | 1 | 0 | 0 | 1 | 0 | 1 | 0 | 2 | 0 | 2 | 0 | 0 | 0 |
| **InfVs** | - | - | - |  | 5 | 0 | 0 | 1 | 1 | 2 | 0 | 0 | 1 | 0 | 1 | 0 | 3 | 0 | 3 | 0 | 0 | 0 |
| **RV** | - | - | - | - |  | 2 | 23 | 5 | 23 | **50** | 2 | 2 | 16 | 7 | **26** | **31** | **45** | **22** | **71** | **22** | 3 | **20** |
| **NL63** | - | - | - | - | - |  | 0 | 0 | 0 | 6 | 0 | 0 | 0 | 1 | 1 | 0 | 0 | 1 | 5 | 2 | 0 | 1 |
| **229E** | - | - | - | - | - | - |  | 0 | 1 | 41 | 0 | 0 | 4 | 0 | 4 | 9 | 10 | 4 | 12 | 3 | 3 | 0 |
| **OC43** | - | - | - | - | - | - | - |  | 4 | 8 | 0 | 0 | 1 | 0 | 1 | 0 | 3 | 1 | 5 | 1 | 0 | 1 |
| **HKU1** | - | - | - | - | - | - | - | - |  | 39 | 0 | 0 | 6 | 1 | 7 | 1 | 9 | 3 | 16 | 6 | 2 | 2 |
| **CoVs** | - | - | - | - | - | - | - | - | - |  | 0 | 0 | 10 | 2 | 12 | 10 | **21** | 9 | **34** | 11 | 5 | 4 |
| **PIV-1** | - | - | - | - | - | - | - | - | - | - |  | 0 | 0 | 0 | 6 | 0 | 1 | 0 | 1 | 1 | 0 | 0 |
| **PIV-2** | - | - | - | - | - | - | - | - | - | - | - |  | 1 | 0 | 4 | 1 | 0 | 0 | 1 | 0 | 1 | 0 |
| **PIV-3** | - | - | - | - | - | - | - | - | - | - | - | - |  | 1 | 34 | 4 | 5 | 5 | 5 | 3 | 3 | 2 |
| **PIV-4** | - | - | - | - | - | - | - | - | - | - | - | - | - |  | 12 | 1 | 2 | 0 | 2 | 4 | 0 | 0 |
| **PIVs** | - | - | - | - | - | - | - | - | - | - | - | - | - | - |  | 6 | 8 | 5 | 9 | 7 | 3 | 2 |
| **MPV A/B** | - | - | - | - | - | - | - | - | - | - | - | - | - | - | - |  | 12 | 2 | 2 | 1 | 7 | 1 |
| **AdV** | - | - | - | - | - | - | - | - | - | - | - | - | - | - | - | - |  | 7 | **28** | 11 | 11 | 0 |
| **BoV** | - | - | - | - | - | - | - | - | - | - | - | - | - | - | - | - | - |  | 10 | 3 | 5 | 1 |
| **RSV A/B** | - | - | - | - | - | - | - | - | - | - | - | - | - | - | - | - | - | - |  | **27** | 10 | **28** |
| **PeV** | - | - | - | - | - | - | - | - | - | - | - | - | - | - | - | - | - | - | - |  | 2 | 0 |
| **EV** | - | - | - | - | - | - | - | - | - | - | - | - | - | - | - | - | - | - | - | - |  | 1 |
| ***S. pneumoniae*** | - | - | - | - | - | - | - | - | - | - | - | - | - | - | - | - | - | - | - | - | - |  |
| **No. of viruses/sample^b^** | | | | | | | | | | | | | | | | | | | | | |  |
| **1virus/sample** | 1 | 0 | 0 | 1 | **92** | 1 | 5 | 2 | 5 | 13 | 3 | 1 | 10 | 0 | 14 | 2 | **7** | 4 | **55** | 0 | 3 | **57** |
| **2** | 1 | 1 | 2 | 4 | **105** | 1 | 14 | 0 | 10 | 25 | 2 | 1 | 6 | 6 | 15 | 23 | **46** | 12 | **73** | 6 | 15 | **54** |
| **3** | 1 | 0 | 2 | 3 | **42** | 2 | 16 | 1 | 12 | **29** | 0 | 1 | 9 | 5 | 14 | 13 | **24** | 15 | **26** | 14 | 9 | **21** |
| **4** | 0 | 0 | 0 | 0 | **24** | 2 | 6 | 2 | 9 | **17** | 1 | 1 | 6 | 1 | 8 | 5 | **9** | 4 | **16** | 13 | 1 | **11** |
| **5** | 0 | 0 | 1 | 1 | **2** | 0 | 0 | 2 | 3 | **4** | 0 | 0 | 2 | 0 | 2 | 1 | **4** | 1 | **5** | 2 | 2 | **4** |
| **6** | 0 | 0 | 0 | 0 | **2** | 0 | 1 | 1 | 0 | **2** | 0 | 0 | 1 | 0 | 1 | 1 | **2** | 1 | **2** | 1 | 0 | **0** |

^a^ Represents the number of possible combinations (virus-virus and/or virus-*S.pneumoniae*). The most frequently detected co-infections are described in bold and colored in blue.

^b^ Describes the number of viruses detected per sample and the possible number of viruses found in co/multiple infection with *S. pneumoniae* (a maximum of 6 viruses were detected per sample).

Abbreviations: InfV-A: *Influenza virus A*, InfV-B: *Influenza virus B*, H1N1: *Influenza virus A (H1N1) swl*, InfVs: *Influenza virus* group, RV: *Rhinovirus*, NL63: *Coronavirus NL63*, 229E: *Coronavirus 229E*, OC43: *Coronavirus OC43*, HKU1: *Coronavirus HKU1*, CoVs: *Coronavirus* group, PIV 1-4: *Parainfluenza viruses 1-4*, PIVs: *Parainfluenza virus* group, MPV A/B: *Metapneumovirus A/B*, AdV: *Adenovirus*, BoV: *Bocavirus*, RSV A/B: *Respiratory Syncytial virus A/B*, PeV: parechovirus genus, EV: enterovirus genus, and *S. pneumoniae*: *Streptococcus pneumoniae*.
